# Intelligent Pattern Recognition Using Distributed Fiber Optic Sensors for Smart Environment

**DOI:** 10.3390/s25010047

**Published:** 2024-12-25

**Authors:** Brian Pamukti, Shofuro Afifah, Shien-Kuei Liaw, Jiun-Yu Sung, Daping Chu

**Affiliations:** 1 Graduate Institute of Electro-Optical Engineering, National Taiwan University of Science and Technology, Taipei 10607, Taiwan; d11119801@mail.ntust.edu.tw (B.P.); d11019802@mail.ntust.edu.tw (S.A.); jysung@mail.ntust.edu.tw (J.-Y.S.); 2 Department of Electronics and Computer Engineering, National Taiwan University of Science and Technology, Taipei 10607, Taiwan; 3 Centre for Photonic Devices and Sensors, Department of Engineering, University of Cambridge, Cambridge CB3 0FA, UK; dpc31@cam.ac.uk

**Keywords:** pattern recognition, deep learning, smart environment

## Abstract

Distributed fiber optic sensors (DFOSs) have become increasingly popular for intrusion detection, particularly in outdoor and restricted zones. Enhancing DFOS performance through advanced signal processing and deep learning techniques is crucial. While effective, conventional neural networks often involve high complexity and significant computational demands. Additionally, the backscattering method requires the signal to travel twice the normal distance, which can be inefficient. We propose an innovative interferometric sensing approach utilizing a Mach–Zehnder interferometer (MZI) combined with a time forest neural network (TFNN) for intrusion detection based on signal patterns. This method leverages advanced sensor characterization techniques and deep learning to improve accuracy and efficiency. Compared to the conventional one-dimensional convolutional neural network (1D-CNN), our proposed approach achieves an 8.43% higher accuracy, demonstrating the significant potential for real-time signal processing applications in smart environments.

## 1. Introduction

Perimeter fences are widely utilized to secure and protect military bases, government installations, petrochemical plants, refineries, and other high-value assets and critical infrastructure from unauthorized access [1,2,3]. Given the extensive lengths of these perimeters, conventional security patrols are often insufficient for adequate coverage. Consequently, implementing a multi-intrusion detection system has become increasingly essential to ensure comprehensive security.

Recent review articles have highlighted the advancements and applications of DFOSs in various fields. For instance, a comprehensive bibliometric analysis by Zhu et al. [4] provides an in-depth overview of signal processing and pattern recognition techniques based on distributed optical fiber sensing. Another study by Liu et al. [5] discusses innovative methods for assessing measurement deviations, which are critical for ensuring the accuracy and reliability of DFOSs in practical applications. These reviews underscore the importance of continuous research and development to address emerging challenges and improve system performance.

High-performance distributed fiber-optic sensors have garnered significant interest in outdoor intrusion detection systems [6,7,8,9,10,11]. These systems can effectively identify and classify intrusion events by leveraging machine learning techniques. Machine learning algorithms enhance the detection capabilities by analyzing patterns and anomalies in the data collected by the sensors. This approach improves the accuracy of intrusion detection and enables the system to adapt to new and evolving threats. Therefore, integrating machine learning is crucial for the successful implementation and reliability of these advanced intrusion detection systems.

Advanced signal recognition and discrimination techniques can significantly enhance intrusion detection systems while maintaining high sensitivity. This can be achieved through various signal processing methods, including simple filtering, adaptive filtering, and parametric, nonparametric, and artificial intelligence (AI) approaches [12,13,14]. Signal classification is central to event recognition and discrimination, involving identifying and extracting unique features from event signals. These signals may represent single events, such as intrusions, rain, and wind, or multiple concurrent events, such as an intrusion during torrential rain. When events occur quickly, it is crucial to employ effective techniques to distinguish events of interest from irrelevant ones.

Several intrusion detection and classification algorithms have recently been proposed [15,16,17,18,19,20,21,22,23,24,25,26]. However, extensive comparisons between deep learning and machine learning for classification have not been explored. Additionally, these studies often compare the probability of detection without evaluating distribution intrusion detection from its algorithms. Marie et al. [17] discussed a hybrid model integrating modern power spectrum estimation (MPSE) and improved gradient neural networks (IGNNs) for event recognition along submarine cables. The model processes vibration signals collected using the technology of phase-sensitive optical time-domain reflectometry (φ-OTDR). The authors reported an average identification accuracy of 97.77% for three categories of data, with specific accuracies of 100%, 96.21%, and 97.11%. The model effectively identifies and classifies collision events on submarine cables in real time, demonstrating robust noise elimination and excellent event recognition capabilities. In addition, researchers [27] used machine learning for underwater image recognition to analyze the environment.

Mi et al. [19] analyzed a vibration pattern recognition algorithm using a merged Sagnac interferometer structure and a multi-layer perceptron neural network (MLP-NN). The pre-processing algorithm retrieves vibration signals, and the MLP-NN classifies these signals into different intrusion patterns. The proposed model achieved 97.6% classification accuracy in tests conducted on a 10 km perimeter fence. The model effectively distinguishes between actual intrusion events and environmental interferences, reducing false alarms and manual workload. Huang et al. [21,22,23] presented a frequency division (FD) all-phase filter bank and a random forest (RF) classifier to detect various types of intrusions. An endpoint detection algorithm was employed to identify the intrusion events. Features extracted from the event data included event fluctuation and zero-crossings rate (ZCR). Their classification algorithm achieved an accuracy of 96.92% for detecting fence-related actions such as kicking, cutting, waggling, climbing, and knocking on the fence. Liu et al. [24] proposed an integrated event discrimination scheme for optical fiber perimeter security systems using empirical mode decomposition (EMD) and kurtosis characteristics combined with a radial basis function (RBF) neural network. This approach improves the recognition rate and variety of intrusion events. The proposed method achieved an average recognition rate of around 85.75% for four common invasive events (climbing the fence, knocking the cable, cutting the fence, and waggling the fence), outperforming frequency domain analysis with an average recognition rate of 68.1%.

An innovative intrusion detection system focused on a pattern recognition scheme for a dual Mach–Zehnder interference (DMZI) distributed fiber perimeter security system has been reported by several researchers [18,25,26]. Lyu et al. [18] proposed a scheme that leverages the gramian angular field (GAF) and convolutional neural network (CNN) to transform one-dimensional intrusion signals into two-dimensional images, which are then analyzed for deep feature extraction. The GAF algorithm enhances the robustness and practicality of the system by being insensitive to power source fluctuations. The proposed method achieves a high recognition accuracy rate of 97.67% for various intrusion events, such as wind, light rain, heavy rain, knocking, impacting, and slapping, with a detection response time of approximately 0.58 s. This approach significantly improves the speed and accuracy of intrusion detection, making it suitable for real-time emergency monitoring applications.

Huang et al. [25] proposed a hybrid feature extraction-based intrusion discrimination scheme for optical fiber perimeter security systems, achieving high classification rates and efficiency. The scheme incorporates various features into a hybrid feature vector, including bandwidth segmentation in the frequency domain, statistical kurtosis, and zero-crossing rate in the time domain. Their experiments showed that the scheme accurately identified four common intrusions—fence climbing, cable knocking, waggling, and fence cutting—with an average recognition rate of over 94% and high efficiency.

Ma et al. [26] developed a probabilistic event discrimination algorithm for fiber optic perimeter security systems, utilizing multiscale permutation entropy and the zero-crossing rate to enhance efficiency and extract intrusion features. A probabilistic support vector machine calculates intrusion probabilities by solving a convex quadratic programming problem. Their experiments show that the algorithm distinguishes six intrusion events with an average recognition rate of 92.68%. This approach provides more detailed intrusion information than traditional methods, reducing decision-making costs and losses from erroneous decisions.

Several researchers have utilized neural networks, hybrid methods, and statistical approaches to achieve high accuracy rates exceeding 90% in intrusion detection. While neural network models are robust, they come with high computational costs due to their complexity. Studies have shown that processing signal data points without converting them to images poses significant challenges in achieving high accuracy. Neural networks like CNN can be fed with one- or two-dimensional datasets, but this approach still faces limitations. Various sensing methodologies have been employed, including φ-OTDR, DMZI, and Sagnac interferometers. Each of these methods has its challenges. For instance, φ-OTDR relies on the backscattering method, which requires the signal to travel nearly twice the distance from the point of intrusion, leading to potential delays and signal degradation. The DMZI approach necessitates a dual setup, increasing the complexity and cost of the system. Sagnac interferometers, although effective, can experience higher attenuation due to the circular fiber configuration.

**Figure 1:**
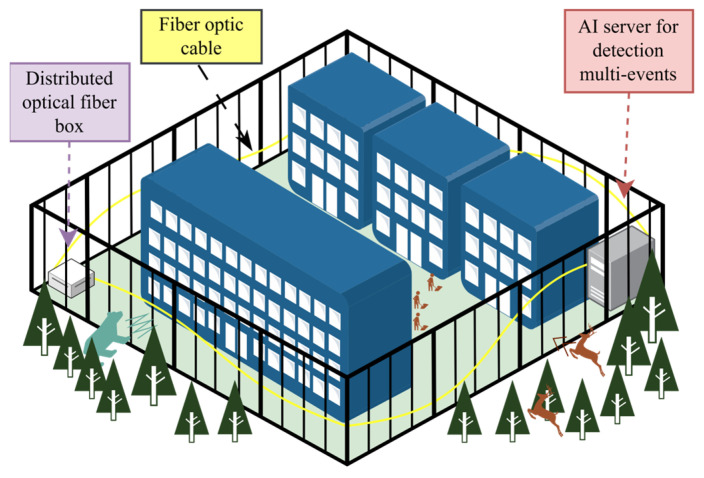
Illustration of the environment in restricted zones.

This paper proposes an innovative deep learning approach for multi-intrusion sensing using one-dimensional time series data with a simpler setup. We introduce an intrusion sensor based on a Mach–Zehnder interferometer (MZI) that employs a deep learning process called time forest neural network (TFNN). The TFNN algorithm uses an interval-based approach for efficient computation, selectively learning from long signals and employing trend detection to enhance the model using gradients. The time series data matrix consists of data points and time intervals, with each data point assigned to a specific category. The algorithm operates with a set number of base estimators and estimators for each base estimator. A key parameter is the minimum interval length considered for feature extraction. The deep learning component of TFNN utilizes dense neural network layers to capture complex patterns and enhance prediction accuracy. In addition, an illustration of the restricted zone for our proposed distributed fiber optic sensor (DFOS) and AI server as part of our TFNN methods can be seen in Figure 1.

This paper is structured as follows: Section 2 describes the experimental setup and model of the TFNN intrusion sensor based on an MZI that employs a machine learning process. Section 3 presents and analyzes the data obtained from the experiments, demonstrating the sensor’s high sensitivity and accuracy. Section 4 compares the results with related studies, highlighting the method’s advantages. Section 5 concludes the research and discusses future work and the implications of the findings.

## 2. Materials and Methods

### 2.1. MZI Optic Fiber Perimeter Intrusion Detection

**Figure 2:**
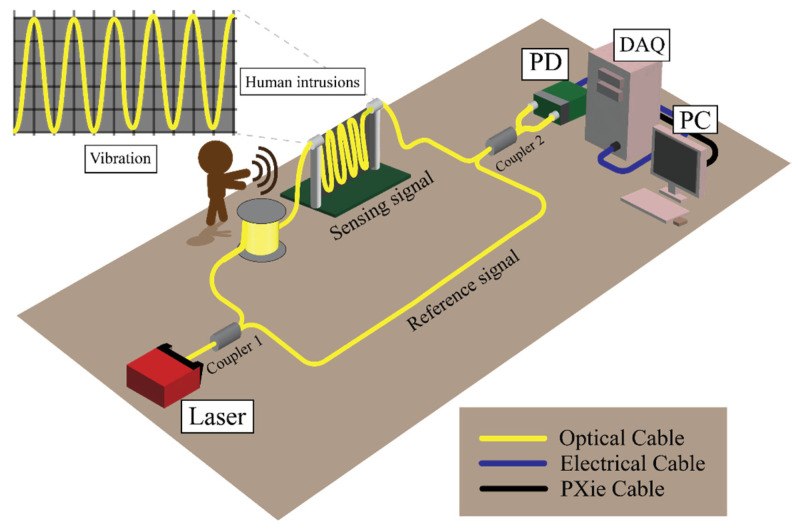
Experimental setup with interferometric sensing.

As depicted in Figure 2, our experimental arrangement utilized a 1530.33 nm distributed feedback (DFB) laser with linewidth 0.1 nm as the light source. An optical coupler with a 3 dB loss (coupler 1) divided the laser’s output into two separate channels: one channel for the reference fiber and the other for the sensing fiber, which extended over one kilometer. This sensing fiber was attached to an iron barrier and exposed to four types of vibrational disturbances. The light from both fibers was then recombined using a second optical coupler (coupler 2). The resulting interference patterns from these intrusions were detected by a balanced photodetector (THORLABS PDB415C), which operates effectively in the 800–1700 nm range and can handle a maximum voltage of 1.55 V across a 50 Ω load. External disturbances can cause changes in the phase of transmitted light waves in the MZI intrusion sensing system:

(1) $$\Delta \phi \left(t\right)=\beta \Delta L+\frac{\partial \beta }{\partial n}\Delta nL+\frac{\partial \beta }{\partial \alpha }\Delta \alpha L,$$

where *L* represents the length of the optical fiber, *β* is the transmission constant of light in the optical fiber, n is the effective refractive index of the optical fiber, and α is the radius of the optical fiber. In this formula, the first term represents the phase change caused by the change of length; the second term represents the phase change caused by the change of refractive index; and the third term represents the phase change caused by the change of optical fiber radius. The fiber coupler converts the change of light in the phase caused by the intrusion event into a change in light intensity. Then, it is output as a voltage by a photodetector so that it can be detected. The expression of output light intensity is defined as follows [28]:

(2) $$I\left(t\right)=I\{1+K\cos\left[\Delta \phi \left(t\right)+\phi_{0}]\right\},$$

where *I* represents the intensity of light, *K* is the influence coefficient of the disturbance signal on light intensity, and *ϕ*_0_ is the initial phase of the light. The output signals from the photodetector were transmitted to a data acquisition (DAQ) system (NI PXIe-1071) via an SMA cable, allowing us to capture time and voltage fluctuations corresponding to the vibrational events. The intrusions analyzed included touching, noise, knocking, and crawling. During the experiment, we captured signals at a sampling rate of 6 kHz to form the dataset. For each type of intrusion, we collected 1000 samples, with each intrusion event taking approximately 10 s to capture. This means that the total time required to create the dataset for each type of intrusion was around 10,000 s. Given that we analyzed four types of intrusions (touching, noise, knocking, and crawling), the total time required to create the dataset was approximately 40,000 s. In addition, our dataset with 1000 samples per event is sufficient, as demonstrated by Ma et al. [29], who used around 393 samples, and Xu et al. [30], who reported an average of 1157 samples per event.

### 2.2. Schematic Model Artificial Intelligence

**Figure 3:**
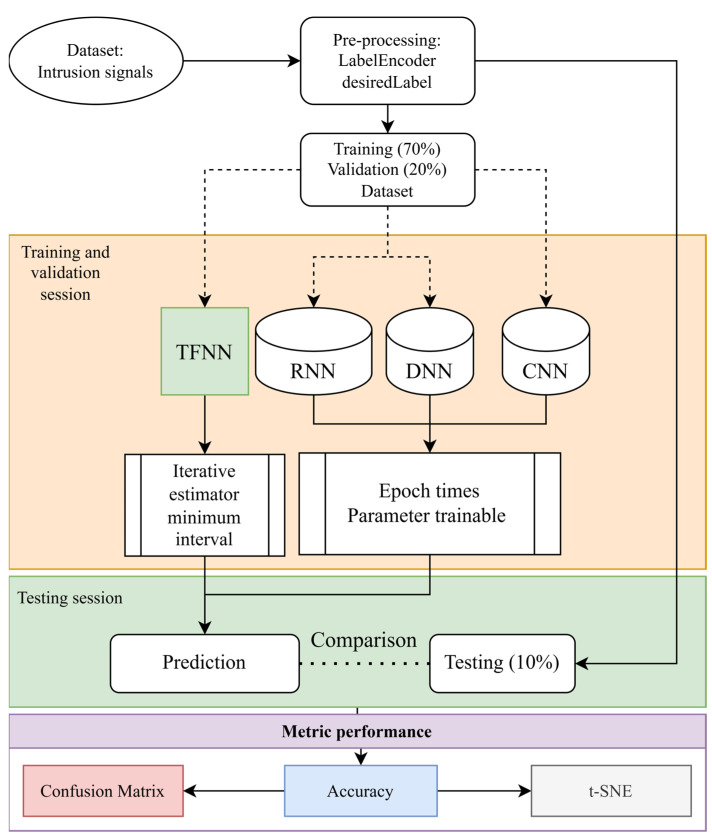
Model comparison experimental workflow.

Figure 3 depicts our research schematic to compare the performance of different neural network models in intrusion signal classification. Once captured by the DAQ system, the signals undergo pre-processing and categorization. The data is then divided into distinct training, validation, and testing sets, with proportions of 70%, 20%, and 10%, respectively. In addition, we used an Intel i9-12900H CPU and an NVIDIA^®^ GeForce RTX 3080 with 16 GB RAM to train our proposed method and neural network models. The TFNN model, inspired by Huang et al. [31], an assembly of decision trees adept at interval-based classification, is our significant contribution to the field. Complementing TFNN in the pipeline are the recurrent neural network (RNN) and dense neural network (DNN), the latter being a more complex iteration of NN with more layers and hidden units for intricate pattern discernment. The one-dimensional convolutional neural network (1D-CNN) is also employed to prioritize and distinguish critical features tailored for signal data. This comprehensive process is meticulously fine-tuned to minimize error intervals, and the performance is rigorously evaluated using accuracy metrics. The culmination of this process is represented in a confusion matrix, providing a clear visual interpretation of the model’s effectiveness in predicting various vibrational events.

### 2.3. Strategy of TFNN

In this study, we propose TFNN using an interval-based approach for efficient computation, as the model learns selectively from long signals and uses trend detection to improve our model using gradient. Let $X\in R^{n\times m}$ be a time series data matrix with dimensions *n* × *m*, where *n* is the number of data points and *m* is the number of time intervals. Additionally, let $y\in R^{n}$ be a class label vector with dimension n, assigning each data point to a specific category. The algorithm works with *B*, the number of base estimators, and *E* is the number of estimators of each base estimator. A key parameter, *l*, denotes the minimum interval length considered for feature extraction. The process begins by selecting a base estimator *C_b_*, and duplicating it to create *C_b,e_* for each interval. The random interval is expressed by

(3) $$I_{b,e}=\left(s_{b,e},t_{b,e}\right),$$

where *s_b,e_* and *t_b,e_* are sampled from the uniform distribution, ensuring that the interval length is at least *l*. For each interval *I_b,e_*, the algorithm extracts features from the data matrix X. The average value within the interval is expressed as

(4) $$\mu_{b,e}=\frac{1}{n}\sum_{i=1}^{n}\frac{1}{t_{b,e}-s_{b,e}}\sum_{j=s_{b,e}}^{t_{b,e}}X_{i,j}.$$

The variability or spread of the values within the interval is also calculated using standard deviation, as shown in

(5) $$\sigma_{b,e}=\sqrt{\frac{1}{n-1}\sum_{i=1}^{n}\left(\frac{1}{t_{b,e}-s_{b,e}}\sum_{j=s_{b,e}}^{t_{b,e}}{\left(X_{i,j}-\mu_{b,e}\right)}^{2}\right)}.$$

The slope of the line connecting the start and end points of the interval, indicating the trend is calculated by gradient, expressed as

(6) $$\alpha_{b,e}=\frac{1}{n}\sum_{i=1}^{n}\frac{X_{i,t_{b,e}}-X_{i,s_{b,e}}}{t_{b,e}-s_{b,e}}\;.$$

These features are combined into a feature vector $F_{b,e}=(\mu_{b,e},\;\sigma_{b,e},\;\alpha_{b,e})$ for each interval. All feature vectors for *E* intervals are then concatenated to form *Z_b_*. The base estimator *C_b_* is trained with the feature matrix *Z_b_* and the class labels *y* as expressed in

(7) $$C_{b}\leftarrow \mathrm{train}\left(Z_{b}\right).$$

For a new data point *x*, features are extracted using the same interval as shown in

(8) $$F_{b,e}\left(x\right)=\left(\mu_{b,e}\left(x\right),\sigma_{b,e}\left(x\right),\;\alpha_{b,e}\left(x\right)\right),$$

where each estimator *C_b,e_* makes a prediction *p_b,e_* (*x*) based on the extracted features. All predictions are combined into a prediction vector *P* (*x*). The final prediction $\hat{y}(x)$ is determined by taking the mode of the prediction *P* (*x*), which represents the most frequently predicted class label by the ensemble of estimators. It leverages the strength of ensemble learning and the informative nature of interval-based features to classify time series data effectively. Using multiple estimators contributes to the robustness and accuracy of the model. The TFNN integrates deep learning by using a simple neural network model for feature extraction and classification. The neural network is composed of dense layers with varying units (32, 16, 4) using ReLU and softmax activations, as shown in Algorithm 1. This deep learning component helps in capturing complex patterns and relationships in the data, further enhancing the model’s predictive performance.
**Algorithm 1.** Time Forest Neural Network1. **Function** TimeForestNN(n_estimators, min_interval):      *estimators* ← [RandomForestClassifier()X n_estimators];     *nn_model* ← create nn_model(); 2. **Function** create nn_model():     model ← Sequential();     **foreach** units ∈ [32, 16, 4] **do**          model.add(Dense(units, activation = units == 4, ’softmax’:’relu’));     **end**     model.compile(optimizer = ’adam’, loss = ’sparse_categorical’);     **return** KerasClassifier(build_fn = lambda: model, epochs = 10); 3. **Function** fit(X,y):     **foreach** rf ∈ estimators do          intervals ← generate intervals(*X.shape* [1]);          features ← extract_features(X, intervals)          rf.fit(features, y)     **end**     nn_features ← extract_features(X, intervals [0])     nn_model.fit(nn_features, y) 4. **Function predict(X):**     rf_predictions ← zeros((X.shape [0], len(estimators)))     **foreach** rf ∈ estimators do          features ← extract_features(X, intervals[i])          rf_predictions[:, i] ← rf.predict(features)     **end**     nn_features ← extract_features(X, intervals [0])     combined_predictions ← apply_along_axis(lambda x: bincount(x).argmax(), axis = 1, arr = rf_predictions) return combined_predictions 5. **Function generate_intervals(series_length):**     intervals ← []     **foreach** _ **do**          start ← randint(0, series_length − min_interval)          end ← randint(start + min_interval, series_length)          intervals.append((start, end))     **end**     **return** intervals 6. **Function extract_features(X, intervals):**     features ← []     **foreach** interval ∈ intervals **do**          start, end ← interval          subset ← X[:, start:end]          mean ← mean(subset, axis = 1)          std ← std(subset, axis = 1)          slope ← (subset[:, −1] − subset[:, 0])/(end − start)          features.append(column_stack([mean, std, slope]))     **end** **return** concatenate(features, axis = 1)

### 2.4. Neural Network Architecture

Pattern recognition encompasses various approaches, including probabilistic methods, machine learning, and deep learning, such as neural networks. Recent research highlights the deep understanding neural networks have in pattern recognition, especially when compared to machine learning or probabilistic approaches. Our study compares different neural network architectures, specifically CNN, DNN, and RNN, to comprehensively evaluate their performance in intrusion detection. CNNs are known for their effectiveness in processing spatial data and extracting features from signal patterns. DNNs provide a more generalized approach with multiple layers, which is suitable for capturing complex patterns in the data. RNNs are particularly effective for sequential data, capturing temporal dependencies, with long short-term memory (LSTM) being a specific implementation within this category. Additionally, we make extensive comparisons among neural network architectures from our proposed model.

**Figure 4:**
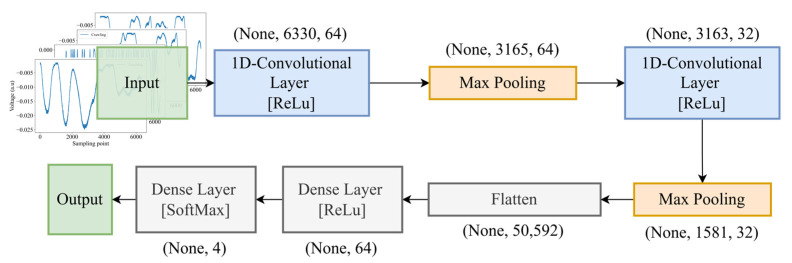
The convolutional neural network (CNN) architecture for intrusion detection.

Different from our proposed model, conventional neural network architectures have several layers, activations, several neurons, and transmission methods, whether forward or backward propagation. First, we trained the CNN model consisting of several layers, starting with a Conv1D layer with 64 filters and a kernel size of 3, followed by a MaxPooling1D layer, as shown in Figure 4. This is succeeded by another Conv1D layer with 32 filters and a subsequent MaxPooling1D layer. The output is then flattened and passed through a Dense layer with 64 units and, finally, a Dense layer with 4 units using the SoftMax activation function. The model has 3,244,644 parameters, all of which are trainable.

**Figure 5:**
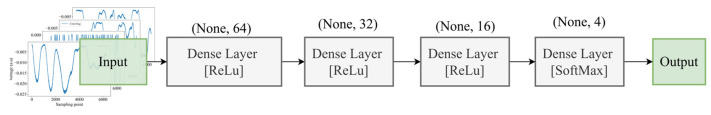
The dense neural network (DNN) architecture for intrusion detections.

The second neural network architecture is the DNN model, which comprises several fully connected layers, as illustrated in Figure 5. It begins with a Dense layer containing 64 neurons, followed by another Dense layer with 32 neurons. A Dense layer with 16 neurons succeeds it and, finally, there is a Dense layer with 4 neurons using the SoftMax activation function. The model processes input data with a shape defined by the input signal from DAQ and employs the ReLU activation function for the hidden layers. The total number of parameters in the model is 407,988, all trainable. This architecture is designed to handle signal data effectively, making it suitable for classification tasks.

**Figure 6:**
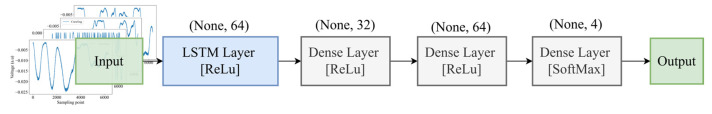
The dense neural network (RNN) architecture for intrusion detections.

Figure 6 shows that the last neural network model is the RNN model, which features an LSTM layer with 64 units, followed by a Dense layer with 32 units. Another Dense layer with 64 units adds this, and, finally, there is a Dense layer with 4 units using the SoftMax activation function. The model is designed to process input data with the shape of a time series data signal, making it suitable for sequential data. The total number of parameters in the model is 21,348, all trainable. This architecture leverages the LSTM layer to capture temporal dependencies in the data, followed by fully connected layers for classification.

In our study, we prioritized the design of neural network models that are both effective and computationally efficient, considering the constraints of our target deployment platforms, such as the Jetson Nano and Raspberry Pi 5. These platforms have limited computational resources, necessitating models with fewer layers and a maximum of 64 neurons per layer. The parameters selected for the TFNN, CNN, DNN, and RNN models were carefully chosen to balance model complexity and computational efficiency for compact devices.

### 2.5. Evaluation Metric

To assess the effectiveness of our strategy, we employed four performance metrics. The first metric is the accuracy score, which measures the ability of the TFNN to generalize and accurately predict each time series signal test. The formula for calculating the accuracy score is as follows:

(9) $$A=\frac{P+N}{P+N+F_{p}+F_{N}},$$

where *P* represents the number of true positive samples correctly identified by the classifier, and *N* denotes the number of true negative samples correctly identified. These are the correct classifications. *F_P_* refers to the number of false positive samples, where the classifier incorrectly labels negative samples as positive. *F_N_* indicates the number of false negative samples, where the classifier incorrectly labels positive samples as negative. These are the incorrect classifications. The precision and F1 scores are additional performance metrics derived from these terms, providing insights into the classifier’s effectiveness in predicting labels.

The second metric is t-distributed stochastic neighbor embedding (t-SNE), a powerful technique for dimensionality reduction and data visualization beneficial for high-dimensional datasets [32]. Mathematically, t-SNE models the pairwise similarities between data points in the high-dimensional space using a probability distribution. It starts by calculating the probability that a data point will be a neighbor to another data point based on its distance, typically using a Gaussian distribution. In the lower-dimensional space, t-SNE aims to preserve these pairwise similarities by modeling them with a t-distribution, which helps maintain the data’s local structure. The algorithm then minimizes the Kullback–Leibler divergence between the two distributions using gradient descent, ensuring that points close in the high-dimensional space (*p*) remain close in the lower-dimensional space (*q*), and those far apart stay distant [33]. We used the Kullback–Leibler algorithm and simplified mathematical express as shown in

(10) $$C=\sum_{i\neq j}p_{ij}\log\left(\frac{p_{ij}}{q_{ij}}\right).$$

This process results in a low-dimensional representation that captures the essential patterns and relationships within the data, making it easier to visualize and interpret complex datasets. By offering qualitative insights through visualization, t-SNE helps to reveal clusters, trends, and anomalies that might not be apparent in the original high-dimensional space. In addition, t-SNE can offer qualitative insights by visualizing the data and the model’s predictions.

## 3. Results

### 3.1. Intrusion Signal Results

**Figure 7:**
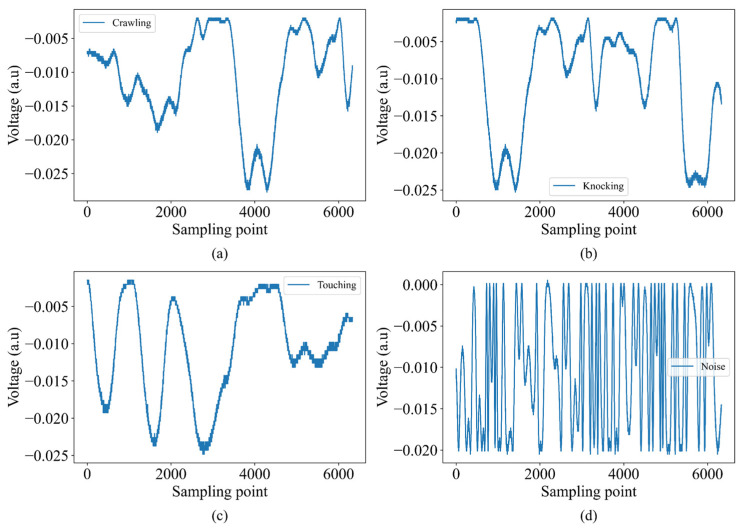
Sample signals obtained by the DAQ system: demonstrating various physical interactions for (**a**) crawling, (**b**) knocking, (**c**) touching, and (**d**) noise.

Figure 7 presents four distinct sample signals obtained by a DAQ system, each corresponding to different physical interactions or disturbances. Figure 7a illustrates a signal labeled “crawling”, showing moderate fluctuations with regular peaks and troughs. Figure 7b displays the “knocking” signal, characterized by similar yet slightly varied peak and trough positions compared to Figure 7a. The “touching” signal in Figure 7c has more pronounced fluctuations, including a significant rise around the 4000-sampling point mark. Finally, Figure 7d shows the “noise” signal, marked by regular voltage changes, indicating no intrusion. These signals are crucial for analyzing and monitoring various events, showcasing the effectiveness of MZI.

**Figure 8:**
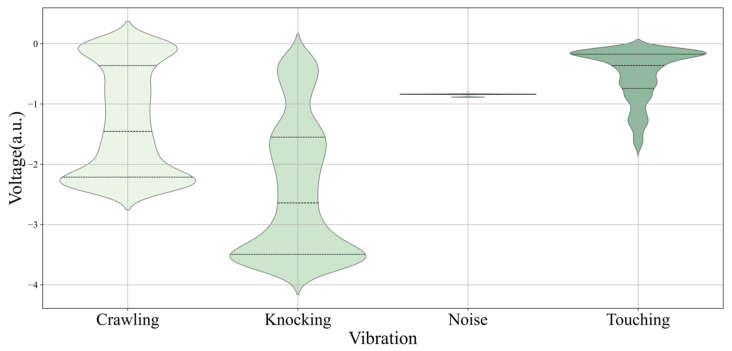
Visual comparison of voltage distributions across four different types of vibrations using violin plots.

Every intrusion has a characteristic value signal with each meaning, standard deviation, and other statistical analysis. Figure 8 shows a violin plot that compares voltage distributions for four intrusion types—crawling, knocking, noise, and touching—to understand our dataset. The “crawling” and “knocking” show similar patterns, with most data points clustering around a central voltage level, showing a common occurrence. The “noise” stands out with a consistent, narrow range, suggesting uniformity in its voltage levels. The “touching” is unique, with a bimodal distribution, revealing two predominant states or behaviors within this category. This visualization is key for finding the distinct electrical signatures associated with each intrusion type, essential for their accurate classification in deep learning applications.

### 3.2. Training Result

We used 2800 samples across the four intrusions for the training set, shuffling the samples to ensure that the model could learn from a random training data pattern. We allocated 800 samples for validation during training to ensure that the model performed well before testing. Finally, we used 400 samples, separate from the training set, to test our model’s performance. Our study aimed to evaluate the performance of various neural network models for intrusion signal classification. The TFNN model employs the number of estimators as its key independent variable, while CNN, DNN, and RNN models use the number of epochs. This distinction is rooted in the fundamental differences between these models and their training processes.

1. TFNN Model: The TFNN utilizes an ensemble method where the number of estimators, or decision trees, is crucial for its performance, as shown in Huang et al. [31]. Each estimator contributes to the model’s overall prediction capability, thus increasing the number of estimators and improving accuracy and computational effort.
2. CNN, DNN, and RNN Models: These models are trained through iterative processes where the number of epochs determines how often the entire training dataset is passed through the model. Each epoch allows the model to learn and adjust its weights, leading to better performance. Therefore, using epochs as the independent variable is appropriate to measure these models’ training progress and performance.

**Figure 9:**
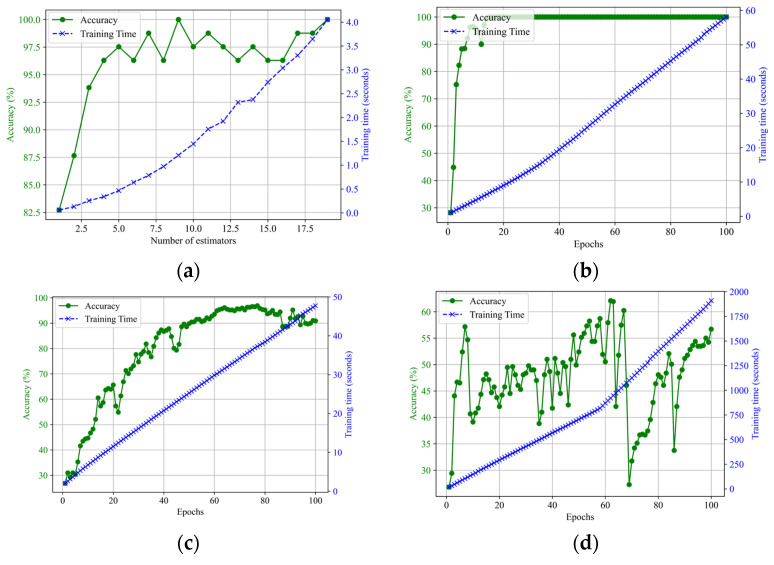
Trend of training accuracy and time vs. number of estimators for (**a**) TFNN and epochs for (**b**) CNN, (**c**) DNN, and (**d**) RNN.

The relationship between the number of estimators, training accuracy, and time for the TFNN model is shown in Figure 9a. As the number of estimators increases, accuracy and training time generally rise. Starting with an accuracy of 82.72% and a training time of 0.0541 s for 1 estimator, the model’s performance improves, reaching 99.99% accuracy at 9 estimators with a training time of 1.2491 s, representing the optimal balance between high accuracy and reasonable training time. The trend continues, reaching 99.99% accuracy at 19 estimators with a training time of 3.9955 s. Figure 9b illustrates the CNN training process over 100 epochs, showing accuracy quickly rising to near 100% and cumulative training time increasing linearly, totaling approximately 58.09 s. Figure 9c depicts the DNN training process over 100 epochs, with accuracy steadily increasing and plateauing near 99.99% and cumulative training time rising gradually to approximately 47.75 s. Figure 9d shows the RNN training process over 100 epochs, with fluctuating accuracy. Still, the cumulative training time generally increases to approximately 1909.21 s, reflecting the computational effort required for neural network architecture.

## 4. Discussion

**Figure 10:**
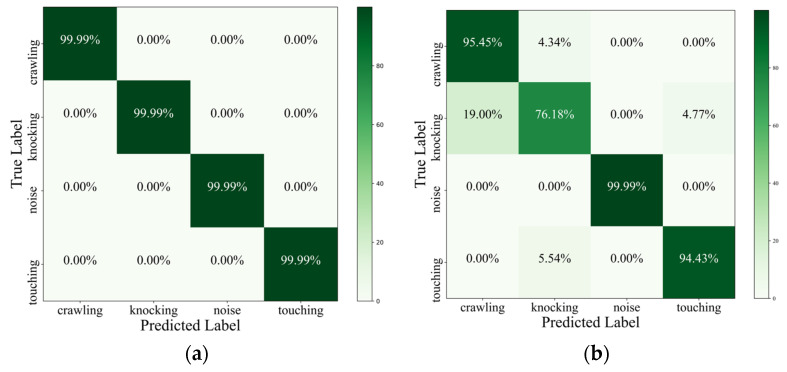

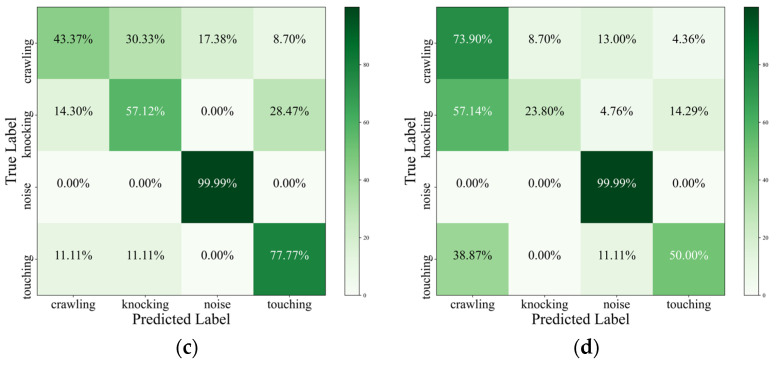
Evaluation of intrusion signal classification using a confusion matrix representation for (**a**) proposed method, (**b**) CNN, (**c**) DNN, and (**d**) RNN.

We evaluate our proposed method alongside other models by employing a confusion matrix, as depicted in Figure 10. The confusion matrix for the TFNN method demonstrates almost perfect classification performance across all four classes: noise, knocking, crawling, and touching, each achieving 99.99% accuracy, as shown in Figure 10a. The CNN model shows strong performance, with high accuracy for most classes: 95.45% for crawling, 76.18% for knocking, 99.99% for noise, and 94.43% for touching, as illustrated in Figure 10b. However, there are some misclassifications, such as 4.34% of crawling instances predicted as knocking and 19.00% of knocking instances predicted as crawling. Figure 10c presents the DNN model’s varying performance, achieving 43.37% accuracy for crawling, 57.12% for knocking, 99.99% for noise, and 77.77% for touching, with notable misclassifications between crawling, knocking, and touching. Figure 10d shows the RNN model’s mixed performance, with 73.90% accuracy for crawling, 23.80% for knocking, 99.99% for noise, and 50.00% for touching, and significant misclassifications, particularly between crawling, knocking, and touching.

Table 1 compares the performance of four models: our method, CNN, DNN, and RNN, across four classes: crawling, knocking, noise, and touching. Our method performs with 99.99% accuracy, precision, recall, and F1 score for all classes. The CNN model shows strong performance, with an overall accuracy of 91.51%, achieving high precision, recall, and F1 scores, particularly for the noise class. The DNN model has a lower overall accuracy of 69.56%, with varying performance across classes, particularly crawling and knocking. The RNN model has the lowest overall accuracy of 61.92%, with significant misclassifications, especially for crawling and knocking, but performs well for the noise class.

**Figure 11:**
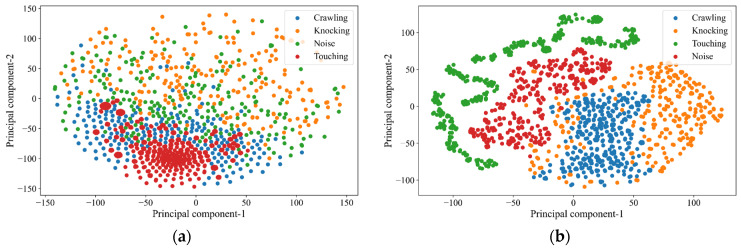
Comparing t-SNE visualizations (**a**) before and (**b**) after applying proposed models.

Figure 11 illustrates the evolution of our deep learning model’s understanding and differentiation of intrusion signals. Initially, the t-SNE plot presents the unprocessed data in a two-dimensional principal part space. Here, the clusters representing different intrusion categories are somewhat indistinct, with considerable overlap, reflecting the data’s raw and unrefined state, as shown in Figure 11a. After applying TFNN, the t-SNE plot transforms, highlighting more pronounced clusters as described in Figure 11b. It indicates the success of the algorithm in pattern recognition and enhancement. The reduced overlap between categories underscores the model’s refined ability to distinguish between events clearly. An increase in cluster density points to the model’s heightened classification precision, bringing similar data points closer. Ultimately, this comparative visualization of the t-SNE plots—before and after deep learning—serves as a testament to the model’s capacity to segregate and interpret complex data, with tighter, well-separated clusters post-training, suggesting robust learning of the nuances among different intrusions like “touching”, “noise”, “knocking”, and “crawling”. The TFNN used in our study is an interval-based classification method inspired by Kang et al. [32], which utilizes dense neural network layers to enhance prediction accuracy. This approach is particularly effective for handling temporal data, focusing on specific intervals to capture the underlying patterns associated with intrusion detection. After approximately six months, we conducted additional experiments at the laboratory by introducing new test datasets to the existing setup and applying our deep learning model without further training. The results indicate that the model remains highly accurate, suggesting its stability.

We compared our method to previous research with similar aims but different approaches concentrating on pattern recognition. Mu et al. [26] report that using the probabilistic method can achieve 92.68% accuracy using DMZI. Huang et al. [25] obtained an average of 94% accuracy using hybrid feature extraction with DMZI. Lyu et al. [18] demonstrated almost the same performance but using a simple setup with a DMZI and converting time series signals to 2D using the GAF algorithm, achieving 97.67% accuracy with a 2D-CNN. Utilizing a single MZI, our method is more efficient and faster than neural network architectures, achieving 99.99% accuracy. This indicates that our enhanced deep learning approach can effectively solve intrusion detection problems as an alternative to traditional neural networks. Given the focus on event pattern recognition, our future work will involve applying longer sensing fibers and improving event localization. Additionally, we are inspired by the experiment methodologies in [18,25,26] and aim to incorporate similar techniques in our model.

While our current manuscript focuses on the advantages of the TFNN algorithm and its comparison with traditional CNN, DNN, and RNN models, future research involves conducting off-site experiments to test the robustness and effectiveness of our intrusion detection system in various external environments. To thoroughly evaluate the system’s resilience, these experiments will include exposure to different noise conditions, such as varying wind speeds and other environmental interferences. Additionally, due to the low complexity and the use of Python programming, future research will implement the system on compact devices such as the Raspberry Pi 5 or Jetson Nano. These experiments will enable us to assess the performance of our model under diverse conditions, thereby providing a comprehensive understanding of its practical applications and potential for real-world deployment. Furthermore, we recognize that Sagnac interferometers offer advantages in robustness for real-world scenarios, particularly in maintaining performance despite cable breaks or other physical disruptions. We will consider incorporating Sagnac interferometers in future experiments to enhance the system’s reliability in practical applications.

## 5. Conclusions

In summary, we conducted extensive experiments using a variety of datasets that included four distinct types of intrusions: crawling, knocking, touching, and noise. Our proposed model showed exceptional performance, achieving 99.99% accuracy across these datasets. Even when visualized using t-SNE, the model maintained clear distinctions between the intrusion types, with only a minor scatter observed between “knocking” and “crawling”. Furthermore, our approach achieved superior performance compared to a conventional neural network model, requiring significantly less computational power and making it more efficient and practical for real-world applications. Our future research will aim to expand upon this work by exploring more than four intrusion types, potentially uncovering even greater insights and applications.

## Figures and Tables

**Table 1:** Comparison of precision, recall, F1 score, and accuracy.

Model	Label	Precision	Recall	F1 Score
	Crawling	99.99%	99.99%	99.99%
Our method	Knocking	99.99%	99.99%	99.99%
Accuracy:	Noise	99.99%	99.99%	99.99%
99.99%	Touching	99.99%	99.99%	99.99%
	Crawling	84.62%	95.45%	89.80%
CNN	Knocking	88.89%	76.18%	82.05%
Accuracy:	Noise	99.99%	99.99%	99.99%
91.51%	Touching	94.44%	94.43%	94.44%
	Crawling	66.67%	43.37%	52.63%
DNN	Knocking	57.14%	57.12%	57.14%
Accuracy:	Noise	82.61%	99.99%	90.48%
69.56%	Touching	63.64%	77.77%	70.00%
	Crawling	47.22%	73.90%	57.63%
RNN	Knocking	71.43%	23.80%	35.71%
Accuracy:	Noise	76.00%	99.99%	86.36%
61.92%	Touching	69.23%	50.00%	58.06%

## Data Availability

Dataset available on request from the authors.
